# Diet Quality, Food Groups and Nutrients Associated with the Gut Microbiota in a Nonwestern Population

**DOI:** 10.3390/nu12102938

**Published:** 2020-09-25

**Authors:** Ángela S. García-Vega, Vanessa Corrales-Agudelo, Alejandro Reyes, Juan S. Escobar

**Affiliations:** 1 Vidarium–Nutrition, Health and Wellness Research Center, Grupo Empresarial Nutresa, Calle 8 sur #50-67, Medellin 050023, Colombia; angela.grc1@gmail.com (Á.S.G.-V.); vcorrales@serviciosnutresa.com (V.C.-A.); 2 Max Planck Tandem Group in Computational Biology, Research Group in Computational Biology and Microbial Ecology (BCEM), Department of Biological Sciences, Universidad de los Andes, Carrera 1 #18-10, Bogota 111711, Colombia; a.reyes@uniandes.edu.co; 3 Edison Family Center for Genome Sciences and Systems Biology, Department of Pathology and Immunology, Washington University School of Medicine, 4523 Clayton Avenue, St. Louis, MO 63108, USA

**Keywords:** food consumption, 24-h dietary recall, gut microbiome, 16S rRNA, short-chain fatty acids, community dwellers, Colombians

## Abstract

Diet plays an important role in shaping gut microbiota. However, much remains to be learned regarding this association. We analyzed dietary intake and gut microbiota in a community-dwelling cohort of 441 Colombians. Diet quality, intake of food groups and nutrient consumption were paired with microbial diversity and composition using linear regressions, Procrustes analyses and a random-forest machine-learning algorithm. Analyses were adjusted for potential confounders, including the five cities from where the participants originated, sex (male, female), age group (18–40 and 41–62 years), BMI (lean, overweight, obese) and socioeconomic status. Microbial diversity was higher in individuals with increased intake of nutrients obtained from plant-food sources, whereas the intake of food groups and nutrients correlated with microbiota structure. Random-forest regressions identified microbial communities associated with different diet components. Two remarkable results confirmed previous expectations regarding the link between diet and microbiota: communities composed of short-chain fatty acid (SCFA) producers were more prevalent in the microbiota of individuals consuming diets rich in fiber and plant-food sources, such as fruits, vegetables and beans. In contrast, an inflammatory microbiota composed of bile-tolerant and putrefactive microorganisms along with opportunistic pathogens thrived in individuals consuming diets enriched in animal-food sources and of low quality, i.e., enriched in ultraprocessed foods and depleted in dietary fiber. This study expands our understanding of the relationship between dietary intake and gut microbiota. We provide evidence that diet is strongly associated with the gut microbial community and highlight generalizable connections between them.

## 1. Introduction

The human gut microbiota is the community of microbial organisms (bacteria, archaea, eukaryotes and virus) living in the dynamic ecosystem of the human gut [1]. This community is highly variable among individuals [2] and has been shown to differ by factors such as geographic origin [3,4], age and sex [5]. The gut microbiota is recognized as an integral part of the human physiology, as some microbial groups provide metabolites that modulate the host immune system to promote health, while others act as opportunistic pathogens eliciting metabolic diseases [6,7,8].

Diet is a strong modulator of the gut microbial community, a fact supported by several lines of evidence. On large phylogenetic scales, it has been shown that the gut microbiota of mammals has adapted to the host diet, with bacterial diversity increasing from carnivory to omnivory to herbivory [9]. Humans harbor a gut microbiota typical of omnivorous mammals [9,10]. The gut microbiota of hunter-gatherers and rural agrarians from Africa and South America, who consume diets rich in plant fibers, has high species diversity, gene richness and complex carbohydrate degradation capabilities [11,12,13,14,15]. Conversely, diminished long-term consumption of dietary fiber and complex carbohydrates results in a progressive and irreversible loss of gut microbiota diversity [16].

Controlled dietary interventions further support the critical role of diet on the diversity and composition of the human gut microbiota. The short-term consumption of diets depleted in fiber and plant-based products increases the abundance of bile-tolerant and pathogenic microorganisms and decreases the levels of microbes that metabolize dietary plant polysaccharides, along with their metagenomic potential and gene expression patterns [17,18]. Improvements in diet quality have also been shown to promote beneficial changes in the gut microbiota, increasing the levels of fiber-degrading bacteria and of genes for microbial carbohydrate degradation linked to short-chain fatty acid (SCFA) metabolism [19,20].

A growing body of evidence indicates that dietary patterns are intimately associated with the human gut microbiota. However, much remains to be learned regarding this association. Studies have mostly focused on controlled dietary interventions. It is not clear how the microbiome pairs with the wider dietary variation encountered in the general population. In addition, studies testing associations between the gut microbiota and diet beyond the USA and Europe are rare. We cannot establish the generalizability of these associations without evaluating diverse human populations. Finally, we ignore which components of the diet are more relevant to modulating gut microbes, be they specific nutrients, whole foods or the overall quality of the diet. In this study, we aim at evaluating associations between different dietary components (diet quality, intakes of foods groups and nutrients) and gut microbiota composition and diversity in a community-dwelling cohort from an understudied population in the midst of the nutritional, epidemiological and demographic transition known as Westernization.

## 2. Materials and Methods

### 2.1. Study Population and Design

Between July and November 2014, we enrolled 459 individuals between 18–62 years of age that were recruited in approximately equal proportions in five large Colombian cities (Bogota, Medellin, Cali, Barranquilla or Bucaramanga), sex (males, females), age groups (18–40 and 41–62 years) and BMI (lean, overweight and obese) (Supplemental Table S1). BMI was measured by a trained member of the research team using Cardinal Detecto DR400C digital scales (St. Webb City, MO, USA) and Seca portable measuring rods (Hamburg, Germany). BMI was calculated as weight (kg)/height squared (m^2^) to classify participants as lean (18.5 ≤ BMI < 25.0 kg/m^2^), overweight (25.0 ≤ BMI < 30.0 kg/m^2^) or obese (BMI ≥ 30.0 kg/m^2^). Individuals were grouped in two age groups to avoid underrepresentation of young adults. Age 40 was arbitrarily chosen as cutoff for these two groups. Previous findings have suggested that the gut microbiota after 65 years is unstable [21]; in consequence, we arbitrarily set age 62 as the upper limit. We also obtained socioeconomic information from the area within each city where an individual had his/her primary residence (six levels according to the Colombian National Administrative Department of Statistics, where level 1 corresponded to the lowest income and 6 to the highest). The socioeconomic distribution of participants was comparable across the five cities, with levels 2, 3 and 4 grouping the great majority of participants in all cities (>80%). All participants were insured by the health insurance provider EPS Sura. We excluded underweight individuals (BMI < 18.5 kg/m^2^), pregnant women, individuals who had consumed antibiotics or antiparasitics less than three months prior to enrollment, and individuals diagnosed with Alzheimer disease, Parkinson disease, or any other neurodegenerative diseases; current or recent cancer (<1 year); and gastrointestinal diseases (Crohn disease, ulcerative colitis, short bowel syndrome, diverticulosis or celiac disease). Of the 459 individuals, we obtained complete diet and microbiota information from 441 of them. Our analytic sample size was therefore 441 individuals.

### 2.2. Ethics

This study was conducted in accordance with the principles of the Declaration of Helsinki, as revised in 2008, and had minimal risk according to the Colombian Ministry of Health (Resolution 008430 of 1993). All of the participants were informed about the study and procedures. Participants were assured of anonymity and confidentiality. Written informed consent was obtained from all the participants before beginning the study. The Bioethics Committee of SIU–Universidad de Antioquia reviewed and approved the protocol and the consent forms (approbation act 14-24-588 dated 28 May 2014).

### 2.3. Dietary Data

Dietary data were collected through 24-h dietary recalls (24-HDR) standardized for the Colombian population, using the National Survey of the Nutritional Situation in Colombia [22] as a reference. The 24-HDR inquired about complete food and beverage descriptions, detailed preparation methods and portion sizes consumed in the previous 24 h. Each participant was personally interviewed at least once by a trained member of the research team. Interviews were randomly distributed on different days of the week. To correctly assess portion sizes and improve accuracy, full-size pictures, geometric figures and real-size food models were used by the interviewers during the surveys. Ninety-four out of 441 participants (21%) were interviewed a second time on a different day of the week to reduce the individual variation between different days of food consumption. Dietary data were introduced into the EVINDI 4.0. software (Medellin, Colombia) [23]. This software was selected because it contained the most complete database of Colombian foods at the time of our analysis; it held the Colombian Food Composition Table [24], the Nutritional Care Center Table 2001 [25] and the USDA’s Food and Nutrient Database for Dietary Studies (FNDDS) 4.1 (2007–2008). No database could be completely updated in this software. In the rare cases where the dietary information of a food was not available in any of these databases (e.g., a new packaged food item), the corresponding information was introduced based upon information provided on the item’s packaging.

Three features were evaluated in each participant’s diet: its quality, the intake of food groups and the intake of nutrients. For diet quality, three indexes were calculated: the first two corresponded to (i) a version of the Healthy Eating Index (HEI) 2015 [26] adapted to our dataset and (ii) a similar index based on the Colombian Food-Based Dietary Guidelines–GABA [27]. (i) The adapted HEI index evaluated dietary quality by favoring the consumption of healthy food groups (i.e., fruits, vegetables, grains, dairy, protein foods, plant proteins, polyunsaturated and monounsaturated fatty acids) and by penalizing the intake of unhealthy foods (i.e., sodium, added sugars and saturated fats). This index was modified to the information available in our dataset (Supplemental Table S2). Portion sizes were calculated as follows: 250 g for a cup and 28.25 g or 29.57 mL for an ounce [26]. The seafood and whole grain components were excluded since the reference databases employed by us did not provide this information. This reduced the maximum points of this index from 100 to 75 (Supplemental Table S2). (ii) The GABA index was similar in its rationale to the adapted HEI but used the Colombian Food-Based Dietary Guidelines–GABA. For this index, instead of calculating cup or ounce equivalents per diet component, we calculated average grams per portion, allowing further calculation of standard values for maximum scores per age and sex [27]. The GABA index was summed across seven food groups and nutrients (Supplemental Table S3). For both the adapted HEI and GABA indexes, a partial score was calculated per food group as the ratio of consumed portions per 1000 kcal. The final score was the sum of all partial scores.

(iii) Additionally, the percentage of calories originating from ultraprocessed foods in the diet of an individual was calculated. Ultraprocessed foods were defined as industrial formulations made from substances extracted from foods (e.g., oils, fats, sugar, starch and proteins), derived from food constituents (e.g., hydrogenated fats and modified starch) or synthesized (e.g., flavor enhancers, colors and several food additives used to make the product more palatable) [28]. For this, each food reported in a given 24-HDR was classified as ultraprocessed or not ultraprocessed. Most of the 14,375 food items reported by the whole cohort (which corresponded to 495 unique items) were easily classified in this way (i.e., 13,358 food items which represented 93% of all reported foods). However, 7% of the food items could be classified into either of the two groups. As an example, the typical Colombian food called *arepa* (grilled patty of soaked, ground kernels of corn or corn flour) is equally common to be bought in its industrialized form (i.e., ultraprocessed) as to be prepared at home (i.e., not ultraprocessed). For the sake of this article, uncertain foods were considered as ultraprocessed. We performed sensitivity analyses by considering these foods as not ultraprocessed. Our conclusions are not affected by this consideration.

For the calculation of the intake of food groups, the reported grams of food items were categorized into food groups following the Colombian Food-Based Dietary Guidelines–GABA [27]. Food groups correspond to a variety of foods with similar nutritional compositions designed to fulfill the standard total caloric requirements of the Colombian population (2650 kcal for males and 2100 kcal for females; [27]). We considered eleven food groups: dairy, meats, eggs, beans, nuts, fruits, vegetables, cereals, tubers, fats and sugars [23,27].

Concerning nutrient intake, calories and macro- and micro- nutrients were calculated from the grams of consumed foods with the EVINDI 4.0. software. Transformation followed the Food Composition Table for the Colombian population [24]. Calories and nutrients were afterwards normalized to reduce both intra- and inter- individual variation by calculating the best linear unbiased predictors (BLUPs) for each nutrient by using information from the second 24-HDRs. BLUPs corresponded to estimated percentile values for usual intakes to transform the data with the most probable intake. BLUPs were calculated with PC-SIDE 1.0. [29].

### 2.4. Gut Microbiota Data

Each participant collected a fecal sample in a sterile receptacle provided by the research team, refrigerated it in a household freezer and brought it within 12 h to a local facility on the same day the 24-HDR interview took place. Stool samples were stored on dry ice and sent to a central laboratory via next-day delivery. Upon receipt, samples were aliquoted and frozen at −80 °C until further analysis.

Gut microbiota diversity and composition were assessed through PCR amplification and sequencing of the V4 hypervariable region of the 16S rRNA gene. A detailed description of the laboratory and bioinformatic procedures used to generate, process and analyze the gut microbiota of participants can be found elsewhere [30]. Briefly, microbial DNA was extracted from the fecal aliquots using the QIAamp DNA Stool Mini Kit (Qiagen; Hilden, Germany). The V4 region of the 16S rRNA gene was amplified with the primers F515 and R806 and sequenced with the Illumina MiSeq v2 platform in a randomized order. To examine the influence of reagent contamination, a negative control (ultrapure water), a DNA extraction blank and a mock community (HM-782D, BEI Resources, Manassas, VA, USA) were included in the analyses. In addition, the reproducibility between sequencing runs was assessed by including replicate samples and determining their differences in operational taxonomic unit (OTU) counts. Amplicons were processed using Mothur v.1.36 following its standard operating procedure, available in November 2015 [31]. OTUs delimited at 97% identity were generated with the average neighbor algorithm and classified using Greengenes 13_8_99 [32]. A relaxed neighbor-joining tree with one representative sequence per OTU was obtained with Clearcut [33] after calculating uncorrected pairwise distances between aligned reads.

Estimates of intra- and inter- subject diversities (alpha and beta diversities, respectively) were calculated with BiodiversityR 2.11 [34] and GUniFrac 1.1 [35]. The Shannon diversity index, the number of observed OTUs and a Shannon evenness index (Jevenness) were calculated as estimates of alpha diversity using Vegan 2.5. Tree-based UniFrac distances were used as estimates of beta diversity. Diversity metrics were obtained on sequence counts rarefied to 3667 sequences per sample, being the number of sequencing reads of the sample with the lowest count.

### 2.5. Statistical Analysis

To understand how diet features varied by participant characteristics, unadjusted means of diet quality indexes, food-group consumption and nutrient intake by sex (male and female) and age group (18–40 and 41–62 years) were calculated. Differences in diet components by variables controlled by design were tested with ANOVA. Afterwards, principal component analyses (PCA) were performed on normalized (z-score) food-group and nutrient intakes.

Differences in gut microbiota alpha diversity across levels of dependent variables were tested with ANOVA, whereas PERMANOVA was performed to test for differences in beta diversity (weighted and unweighted UniFrac distances). In addition, the 100 most abundant OTUs with median relative abundance across all individuals ≥0.01% were extracted; these OTUs represented 80 ± 12% of the 16S rRNA gene reads in our dataset.

Dietary information and gut microbiota alpha diversity were paired by using multivariable-adjusted linear regressions. To address potential confounding associations, multivariable models included the participants’ city of residence, sex, age group, BMI and socioeconomic level. Models were adjusted by city because this is one of the main drivers of gut community structure in this cohort [36]. Likewise, sex and age are notable contributors of both microbiota diversity [5] and diet (see our results below). BMI has also been shown to affect gut microbiota composition and diversity [36,37], while the household socioeconomic status is strongly linked with its purchasing power and likely associated with food choices. These models were run on normalized diet variables as well as on the first three components of food-group and nutrient PCAs.

Associations between diet components and gut microbiota beta diversity (weighted and unweighted UniFrac distances) were tested by using Procrustes analysis with 10,000 permutations. In addition, a regression-based random-forest machine-learning algorithm was used to pair the relative abundance of each of the 100 most abundant OTUs with multivariable-adjusted levels of the different diet components evaluated here: quality indexes, food-group and nutrient intakes. Random-forest models were also obtained for the first three components of the aforementioned PCAs. This modeling used a decision tree-based approach that accounted for nonlinear data and included an internal cross-validation to prevent overfitting. For each tree, two-thirds of the samples were randomly selected for training the model and one-third for testing [38]. These models classified OTUs by their degree of association with a given diet variable and were sorted according to the importance of each given OTU to the selected models. This importance was determined by the increase in the mean square error when a given OTU was not included in the model. The selected models were those that maximized the explained variance. The direction of associations between OTU abundance and the diet variable were determined using Spearman correlation coefficients. Random-forest models were generated with the randomForest 4.6 package of R 3.6.1 with 50,000 trees.

### 2.6. Data Availability

Raw DNA reads (FASTQ) are available at the Sequence Read Archive at NCBI under BioProject PRJNA417579. The R code to reproduce statistical analyses is available at https://github.com/vidarium/diet_microbiota_MiSalud1.0.

## 3. Results

The analyzed dataset was obtained from an urban community-dwelling cohort of 441 Colombian adults, on which diet quality and intakes of food groups and nutrients were assessed through 24-HDR, alongside with the gut microbiota composition and diversity, which were evaluated through 16S rRNA gene sequencing. Linear regressions, Procrustes analyses and a regression-based random-forest machine-learning algorithm were used to pair diet and gut microbiota. Importantly, all analyses were adjusted for measured confounders, including the city where participants originated, sex, age group, BMI and socioeconomic status.

### 3.1. Dietary Analysis

The average caloric intake of the studied population was 2135 kcal for males and 1750 kcal for females (Table 1). These values were close to the average caloric intake per sex reported for the Colombian population (males: 2197 kcal/day, females: 1838 kcal/day) [39]. The caloric intake was not associated with the participants’ socioeconomic level (ANOVA: F_5, 427_ = 0.27, *p* = 0.93) but depended on sex (F_1, 427_ = 101.22, *p* < 0.0001), age group (F_1, 427_ = 18.86, *p* < 0.0001), the city of origin (F_4, 427_ = 3.34, *p* = 0.01) and, marginally, BMI (F_2, 427_ = 2.88, *p* = 0.06).

In terms of diet quality, on average, the studied population had an adapted HEI of 40/75 and a GABA index of 27/70, meaning that, overall, individuals consumed about half of the foods and portions recommended for a healthful and nutritionally adequate diet. This is in agreement with previous reports in this population [40]. Additionally, 35% of the subjects’ total caloric consumption was provided, on average, by ultraprocessed food items. Of note, it was observed that females and middle-aged individuals had better diet quality than males and younger individuals (Table 1).

Next, each ingested food was classified into one of eleven food groups: dairy, meats, eggs, beans, nuts, fruits, vegetables, cereals, tubers, fats and sugars. Consumption of all food groups was lower than the amounts recommended for the Colombian population (Supplemental Table S4). In terms of total grams, the main consumed food groups were cereals, sugars, fruits and dairy (Table 1). Food-group intake differed by sex and age group: males and young individuals had significantly higher intake of meats, beans, cereals, tubers, fats and sugars than females and middle-aged subjects. Interestingly, in several food groups recommended for healthy diets such as dairy, nuts, fruits and vegetables, the trend was inverted with higher consumption in females and middle-aged individuals, although the trend was only statistically significant for fruits and vegetables by age group (Table 1).

**Figure 1:**
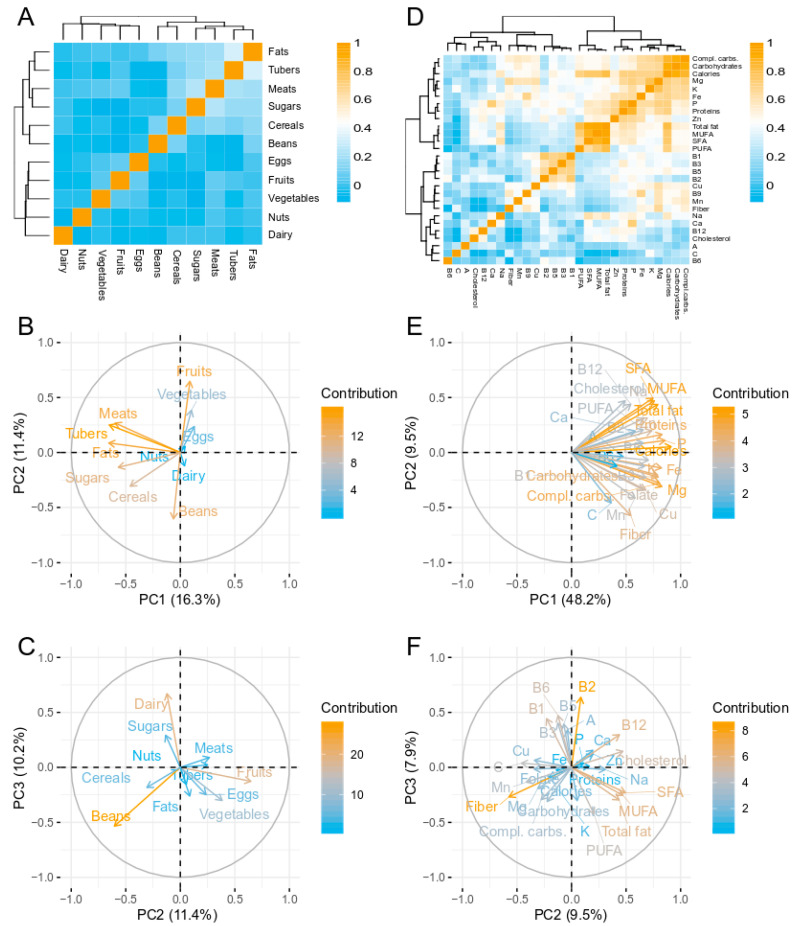
Diet features in the studied population. (**A**) Heatmap showing Pearson’s product moment correlations between pairs of food groups. Dendrograms obtained by hierarchical Ward-linkage clustering. (**B**,**C**) Principal component analysis (PCA) projecting the intake of food groups on the first three components. (**D**) Heatmap showing Pearson’s product moment correlations between pairs of nutrients. (**E**,**F**) PCA projecting the intake of nutrients on the first three components.

A PCA was performed in order to reduce the dimensionality in food-group data and decrease the effect of intercorrelated variables (Figure 1A). The first component (PC1) captured 16.3% of the variance and was associated with food groups that dominate the Colombian plate: meats, tubers, fats, cereals and sugars. PC2 provided information about food groups rich in dietary fiber, separating fruits and vegetables from beans; this component explained 11.4% of the variance (Figure 1B). PC3 separated dairy and food groups rich in fiber (beans, vegetables and fruits), and explained 10.2% of the variance (Figure 1C).

Food groups were broken down further according to their nutrient contents. Males and younger individuals consumed significantly more macro- and micro- nutrients than females and middle-aged subjects (Table 1). There were some exceptions to this pattern: fiber, calcium and vitamin A tended to be consumed in higher amounts in middle-aged females, whereas vitamin C was consumed in significantly higher amounts in middle-aged rather than young individuals, for both males and females (Table 1).

More than 70% of the individuals had an acceptable macronutrient intake distribution, according to the Colombian Energy and Nutrient Intake Recommendations [41], meaning that 50–65% of total calories were obtained from carbohydrates, 14–20% from proteins and 20–35% from fats. In contrast, only 15% of the middle-aged females had an adequate intake of dietary fiber, while 95% of younger females and almost all males consumed inadequate amounts of it. Nearly all micronutrients were consumed in deficient amounts, with the lowest adequacy for calcium, potassium, magnesium, and vitamins B1, B5, B6 and B9 (folate). Due to the difference by sex in the nutrient requirement, zinc was found to be deficient in almost all males and in excess in 100% of females. Phosphorus, total iron, copper, manganese, and vitamins A, C and B12 were consumed in adequate or high amounts by most of the cohort (Supplemental Table S4).

Given that several macro- and micro- nutrients were strongly intercorrelated (Figure 1D), an additional PCA was performed on normalized nutrient intake. It was found that PC1, which explained 48.2% of the variance, was driven by the amount of consumed calories, macro- and micro-nutrients, as all these variables were positively correlated with this component (Figure 1E). Interestingly, PC2, which explained 9.5% of the variance, provided information about the sources of nutrients: negative values were indicative of nutrients obtained mainly from plant-food sources (e.g., fiber, carbohydrates, vitamin C, folate) whereas positive values were associated with nutrients obtained mainly from animal-food sources (e.g., vitamin B12, cholesterol, saturated fatty acids) (Figure 1E). PC3 discriminated the intake of vitamins of the B complex from macronutrients like fiber, carbohydrates and fats (Figure 1F).

### 3.2. Gut Microbiota Analysis

First, 16S rRNA gene sequencing of the participants’ stools resulted in a total of 33,448 ± 17,131 reads per sample (median = 28,561, range: 3667–102,660) that were rarefied at 3667 reads for assessment of community composition and diversity. A total of 2505 OTUs were observed after rarefaction. Parallel sequencing of a mock community revealed a mean sequencing error rate of 0.12%, and sequencing of replicate samples in different runs indicated that the difference between sequencing runs was minor (maximum sequence count difference between OTUs of replicate samples on rarefied data for all replicates = 85 reads; overall median differences = 0 reads).

**Figure 2:**
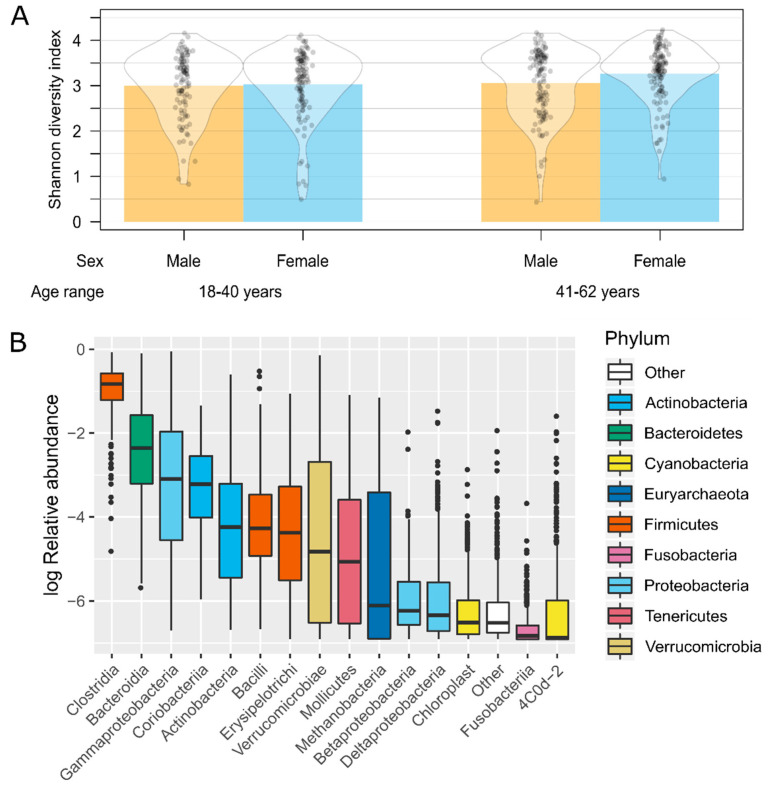
Gut microbiota diversity and composition of the studied population. (**A**) Distribution of alpha diversity (Shannon diversity index) by sex and age group. (**B**) Taxonomic profile at the class level. Classes with a median relative abundance equal to zero were combined into “Other”. The color code corresponds to the taxonomic classification at the phylum level.

The Shannon diversity index, which quantified differences in gut microbiota diversity within individuals (i.e., alpha diversity), was significantly higher in females than males (ANOVA: F_1, 427_ = 3.99, *p* = 0.046) and in middle-aged than younger individuals (F_1, 427_ = 6.38, *p* = 0.012) (Figure 2A). It also differed by city of origin (F_4, 427_ = 9.60, *p* < 0.0001) and BMI (F_2, 427_ = 3.69, *p* = 0.026) but did not change according to socioeconomic level (F_5, 427_ = 1.83, *p* = 0.11). The gut microbiota of the studied population was dominated by *Firmicutes* and *Bacteroidetes* phyla, in particular by the *Clostridia* and *Bacteroidia* taxonomic classes, followed by other groups in lower abundances (Figure 2B). Tree-based weighted UniFrac distances, which quantified differences in gut microbiota diversity between individuals (i.e., beta diversity), differed by the participants’ city of origin (PERMANOVA: R^2^ = 0.073, *p* = 0.001), sex (R^2^ = 0.012, *p* = 0.001), socioeconomic level (R^2^ = 0.015, *p* = 0.024) and BMI (R^2^ = 0.009, *p* = 0.002), but were not associated with the participants’ age group (R^2^ = 0.003, *p* = 0.17).

### 3.3. Associations between Diet and Gut Microbiota

Diet and microbiota data were paired to assess how the intake of the studied population affected the composition and diversity of the gut microbial community. To this end, linear regressions, Procrustes analyses and random-forest regressions were employed. The random-forest models used the relative abundance of the 100 most abundant OTUs of the dataset and classified them by their importance for each individual model (Supplemental Table S5). We did not evaluate most other, rarer OTUs in the dataset since they were not expected to be confidently associated with diet, as they were detected in very low abundances (<0.01%) and were more susceptible to sequencing artifacts.

**Figure 3:**
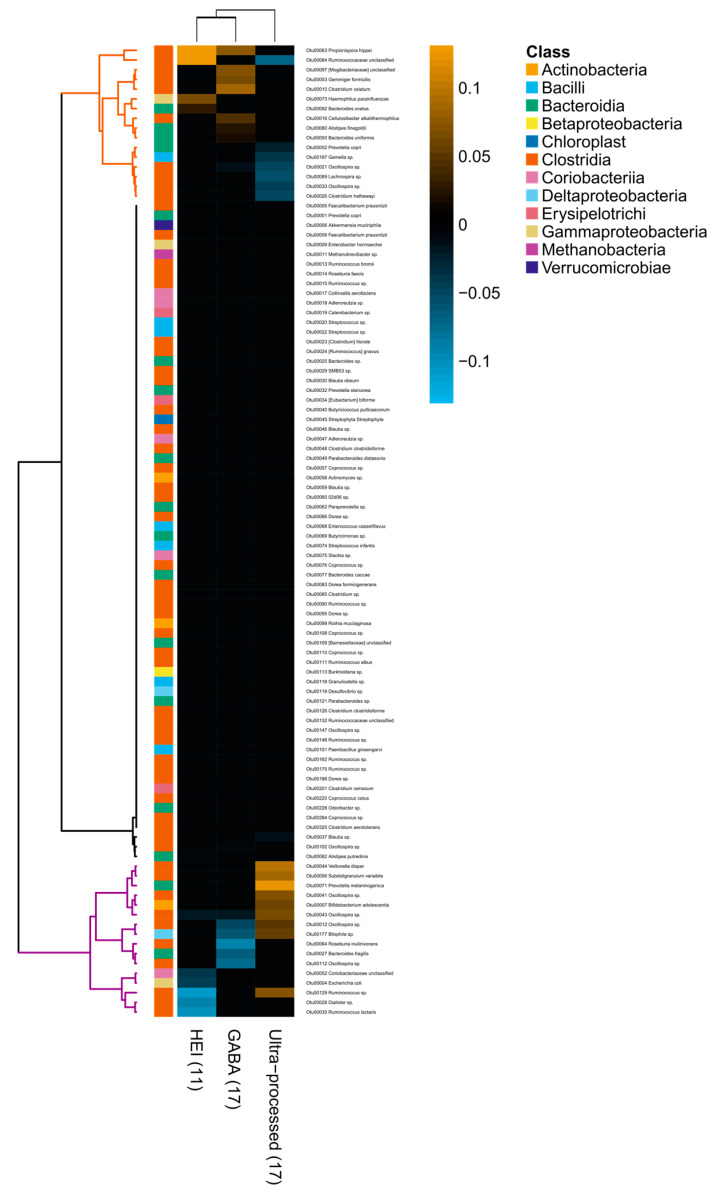
Heatmap showing Spearman’s correlation coefficients between operational taxonomic unit (OTU) relative abundance and diet quality. The set of OTUs associated with multivariable-adjusted diet quality indexes were obtained with a regression-based random-forest machine-learning algorithm. Dendrograms obtained by hierarchical Ward-linkage clustering. The colored branches of the dendrogram are for illustrative purposes: brown branches highlight OTUs associated with diets of high quality, while purple branches highlight OTUs associated with diets of low quality. The taxonomic classification at the class level of each OTU is noted at the left side of the heatmap. Values in parentheses next to quality indexes indicate the number of OTUs selected by the random forest. HEI = adapted Healthy Eating Index 2015; GABA = Colombian Food-Based Dietary Guidelines.

In terms of diet quality, the random-forest regressions identified two well-differentiated microbial communities: one community pairing with higher HEI and GABA scores and lower intake of ultraprocessed foods; and the other pairing with the inverse dietary pattern (Figure 3). The community associated with diets of high quality included OTUs from SCFA-producing *Clostridia*, such as *Propionispora hippei*, an unclassified *Ruminococcaceae*, *Gemmiger formicilis*, *Cellulosibacter alkalithermophilus*, *Oscillospira* sp. and *Lachnospira* sp. It also included OTUs from *Bacteroidetes* like *Bacteroides ovatus*, *Bacteroides uniformis*, *Alistipes finegoldii* and *Prevotella copri*, as well as the opportunistic pathogen *Haemophilus parainfluenzae*. On the other hand, the community pairing with diets of low quality and high intake of ultraprocessed foods included OTUs from bile-tolerant *Bilophila* sp. and opportunistic pathogens such as *Escherichia coli*, *Bacteroides fragilis* and *Prevotella melaninogenica*. It also included SCFA producers such as *Subdoligranulum variabile*, *Oscillospira* sp., *Bifidobacterium adolescentis*, *Roseburia inulinivorans*, *Ruminococcus* sp. and *Ruminococcus lactaris*. No association between any of the three employed quality indexes and the gut microbiota alpha diversity was found (HEI: F_1, 426_ = 0.46, *p* = 0.50; GABA: F_1, 426_ = 0.44, *p* = 0.50; ultraprocessed foods: F_1, 426_ = 0.01, *p* = 0.91), nor were associations significant between diet quality and beta diversity (Procrustes rotation = 0.04, *p*-value based on 10,000 permutations = 0.51).

**Figure 4:**
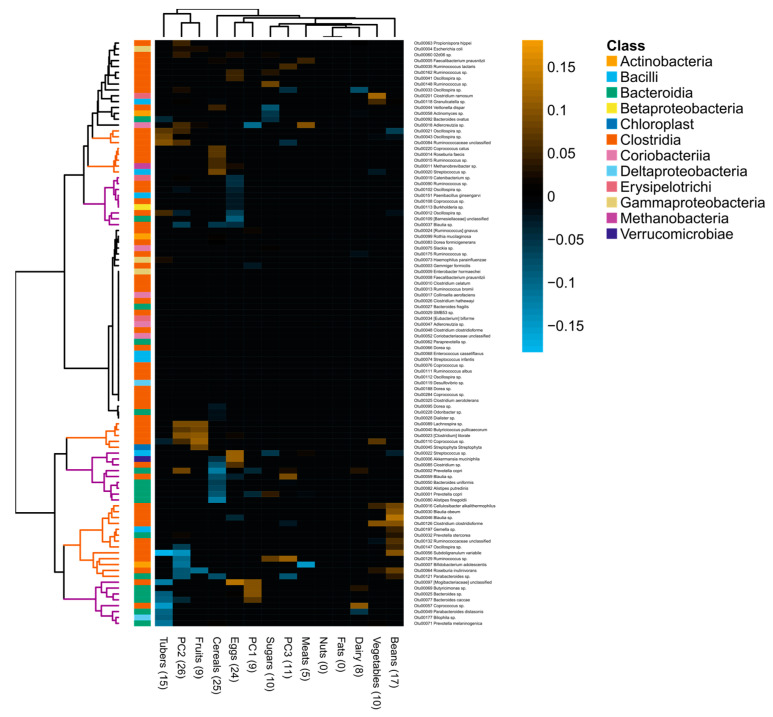
Heatmap showing Spearman’s correlation coefficients between OTU relative abundance and food-group intake. The set of OTUs associated with multivariable-adjusted food-group intake was obtained with a regression-based random-forest machine-learning algorithm. We also included the first three components of the food-group PCA. Dendrograms obtained by hierarchical Ward-linkage clustering. The colored branches of the dendrogram are for illustrative purposes: brown branches highlight OTUs associated with plant-derived food groups, while purple branches highlight OTUs associated with animal-derived food groups. The taxonomic classification at the class level of each OTU is noted at the left side of the heatmap. Values in parentheses in the *x*-axis indicate the number of OTUs selected by the random forest.

The Procrustes analysis indicated that a subject’s food-group intake correlated with the microbiota structure, according to tree-based weighted UniFrac distances (correlation in a symmetric Procrustes rotation = 0.15, *p*-value based on 10,000 permutations = 0.0016). Random-forest regressions detailed this association (Figure 4). They showed that communities of SCFA-producing *Clostridia* were mainly positively associated with intake of plant-derived food groups, rich in dietary fiber, such as fruits and vegetables (i.e., positive PC2) as well as beans (i.e., negative PC2). These included OTUs from *Oscillospira* sp., unclassified *Ruminococcaceae*, *Coprococcus catus*, *Roseburia faecis*, *Ruminococcus* sp., *Lachnospira* sp., *Butyricicoccus pullicaecorum*, *Coprococcus* sp., *Cellulosibacter alkalithermophilus*, *Clostridium clostridioforme*, *Subdoligranulum variabile* and *Roseburia inulinivorans*, among others. The methanogen archaea *Methanobrevibacter* sp., *Bifidobacterium adolescentis*, an unclassified *Streptophyta* and one OTU of *Streptococcus* sp. were also associated with plant-derived food groups (Figure 4).

Interestingly, it was found that a different OTU of *Streptococcus* sp., along with *Akkermansia muciniphila*, bile-tolerant *Bilophila* sp., *Prevotella copri*, *Bacteroides* spp., *Alistipes putredinis*, *Alistipes finegoldii*, *Butyricimonas* sp., *Parabacteroides distasonis* and the opportunistic pathogen *Prevotella melaninogenica*, were associated with increased intake of animal-derived foods, like eggs and dairy, and reduced intake of tubers and cereals. Also, a large group of microbes that were negatively associated with egg consumption was found, including *Catenibacterium* sp., *Ruminococcus* sp., *Oscillospira* sp., *Paenibacillus ginsengarvi*, *Coprococcus* sp., *Burkholderia* sp., an unclassified *Barnesiellaceae* and *Blautia* sp. Note that food groups were not associated with gut microbiota alpha diversity (all *p*-values from ANOVA >0.10), and that no OTUs were associated with the consumption of nuts or fats (Figure 4).

**Figure 5:**
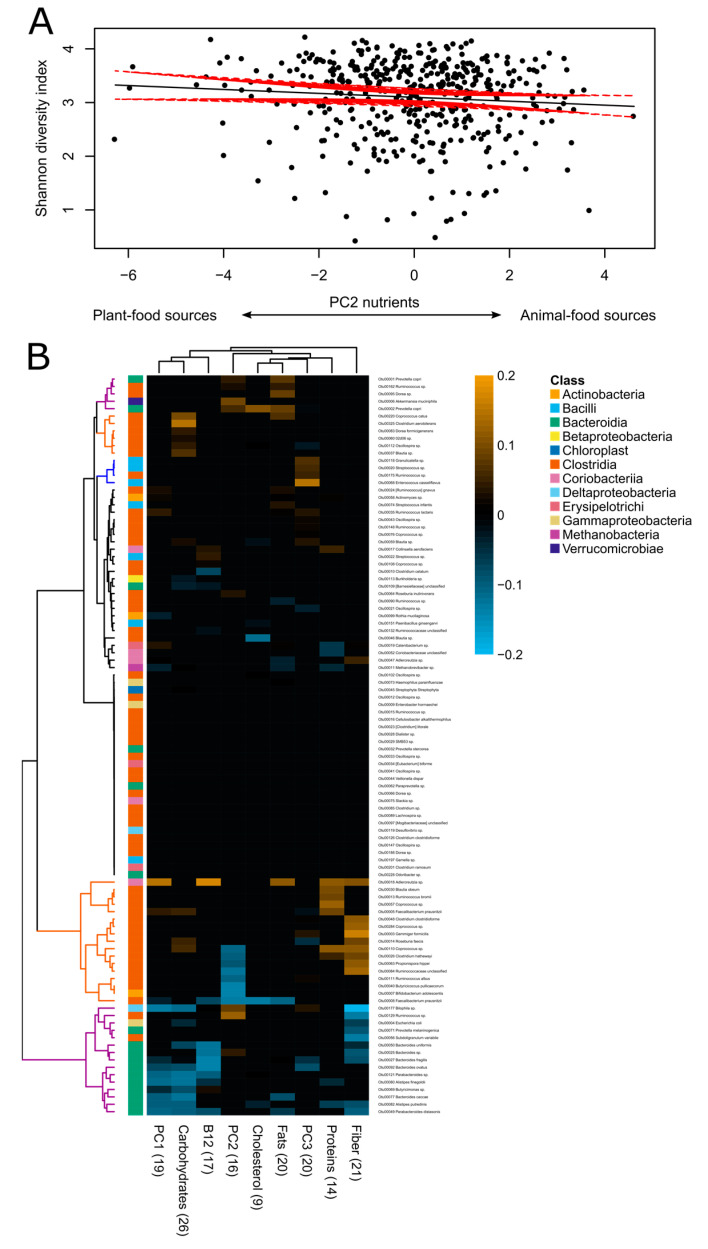
Associations between nutrient intake and gut microbiota. (**A**) Correlation between the gut microbiota alpha diversity (Shannon diversity index) and nutrient intake (regression line with 95% confidence intervals). PC2 (*x*-axis) provides information about the sources of nutrients: negative values indicate nutrients obtained mainly from plant-food sources, whereas positive values are associated with nutrients obtained mainly from animal-food sources. (**B**) Heatmap showing Spearman’s correlation coefficients between OTU relative abundance and nutrient intake. The set of OTUs associated with multivariable-adjusted nutrient intake were obtained with a regression-based random-forest machine-learning algorithm. We also included the first three components of the nutrient PCA. Dendrograms obtained by hierarchical Ward-linkage clustering. The colored branches of the dendrogram are for illustrative purposes: brown branches highlight OTUs associated with nutrients mainly obtained from plant-food sources, purple branches highlight OTUs associated with nutrients mainly obtained from animal-food sources and blue branches highlight OTUs associated with PC3 (i.e., vitamins of the B complex). The taxonomic classification at the class level of each OTU is noted on the left side of the heatmap. Values in parentheses in the *x*-axis indicate the number of OTUs selected by the random forest.

The analysis at the nutrient level indicated that individuals obtaining nutrients mainly from plant-food sources (i.e., negative PC2) tended to have a more diverse gut microbiota than individuals obtaining them from animal-food sources (i.e., positive PC2) (ANOVA: *p* = 0.06; Figure 5A). Nutrient intake was also associated with gut microbiota beta diversity (weighted UniFrac: correlation in a symmetric Procrustes rotation = 0.16, *p*-value based on 10,000 permutations = 0.004). Random-forest regressions identified distinct communities associated with principal components and macro- and micro- nutrients. A community of 17 OTUs, mostly from the *Clostridia* class, was associated with high intake of proteins and fiber, and low PC2 values (i.e., higher intake of nutrients obtained from plant-food sources). Many of these microbes are known SCFA producers, including *Ruminococcus bromii*, *Coprococcus* sp., *Faecalibacterium prausnitzii*, *Clostridium clostridioforme*, *Gemmiger formicilis*, *Roseburia faecis*, *Clostridium hathewayi*, *Propionispora hippei*, unclassified *Ruminococcaceae*, *Ruminococcus albus*, *Butyricicoccus pullicaecorum*, and *Bifidobacterium adolescentis*. Other *Clostridia* were associated with high intake of carbohydrates: *Coprococcus catus*, *Clostridium aerotolerans*, *Dorea formicigenerans*, *02d06* sp., *Oscillospira* sp. and *Blautia* sp. (Figure 5B).

On the other hand, a community of 15 OTUs was found to be associated with low intake of fiber and carbohydrates, and high PC2 (i.e., higher intake of nutrients obtained from animal-food sources). This included opportunistic pathogens such as *Escherichia coli*, *Bacteroides fragilis* and *Prevotella melaninogenica*, in addition to many *Bacteroidia*, including bile-tolerant *Bilophila* sp., *Bacteroides* spp., *Parabacteroides* spp., *Alistipes finegoldii*, *Alistipes putredinis* and *Butyricimonas* sp. Another community of bacteria including *Prevotella copri*, *Akkermansia muciniphila*, *Ruminococcus* sp. and *Dorea* sp. was associated with high PC2, dietary cholesterol and fats. Finally, a small group of OTUs including *Granulicatella* sp., *Streptococcus* sp., *Enterococcus casseliflavus* and *Ruminococcus* sp. (Figure 5B) was associated with high PC3 (i.e., vitamins of the B complex).

## 4. Discussion

Using distinct statistical analyses adjusted for potential confounders, we demonstrated that diet quality as well as intakes of food groups and nutrients exhibited meaningful associations with the gut microbiota of a population that is in the middle of the nutrition transition known as Westernization. Participants consuming diets of higher quality, richer in plant-derived foods and fiber tended to have a more diverse gut microbiota and increased levels of beneficial SCFA-producing bacteria, mainly from the *Clostridia* taxonomic class (*Firmicutes*), despite the fact that intake of fiber, fruits and vegetables was deficient in most of the studied participants. This suggests that the enrichment in SCFA-producing bacteria and the ecological service of providing SCFAs to the human host are achieved even with moderate consumption of dietary fiber (in our case, about 18 g/day). A threshold of fiber intake might thus exist above which the purported beneficial effects are obtained. We anticipate that such effects will be stronger in individuals consuming adequate amounts of fiber (i.e., >14 g/1000 kcal).

Mounting evidence suggests that this is a general pattern through which the human gut microbiota is connected to diet. Populations with traditional lifestyles consuming diets with abundant plant polysaccharides have a microbiome enriched in carbohydrate active enzymes, pathways associated with the degradation of dietary fiber and production of SCFAs [11,12,13,14,15]. In Western cohorts, it has also been shown that individuals consuming diets of high quality, i.e., rich in plant-derived foods and with lower intake of sugars, saturated fats and animal-derived foods, have a diverse and beneficial microbiota, with overrepresentation of SCFA-producing enzymes and SCFA-producing bacteria [18,19,20,42,43]. SCFAs, especially butyrate, and SCFA-producing bacteria are beneficial to the human host. They have anti-inflammatory properties [44], nourish colonocytes [45] and play roles in the development of the intestinal epithelial barrier [46,47] and in immune responses [48].

On the other end of the continuum, our results indicate that individuals with higher consumption of ultraprocessed foods and lower intake of fruits, vegetables and fiber had a microbiota described as inflammatory enriched in bile-tolerant and putrefactive microorganisms, such as *Bilophila*, *Alistipes* and *Bacteroides*, along with opportunistic pathogens such as *Escherichia coli*, *Bacteroides fragilis* and *Prevotella melaninogenica*. Remarkably, similar results have been found in well-controlled dietary interventions. David et al. [17] showed that the short-term consumption of diets composed entirely of animal products increased the abundance of bile-tolerant and putrefactive microorganisms (e.g., *Bilophila wadsworthia*, *Alistipes putredinis* and *Bacteroides* sp.), in addition to the proliferation of *Alphaproteobacteria* (e.g., *Escherichia*, *Raoultella* and *Moraxellaceae*). Likewise, O’Keefe et al. [18] showed that switching rural Africans to a “Western” high-fat, low-fiber diet resulted in increased bile-acid synthesis and higher abundance of *Bilophila wadsworthia*, a sulfite-reducing bacterium whose production of hydrogen sulfide leads to acute inflammation of the intestinal tissue [49,50].

The opportunistic pathogens enriched in the gut microbiota of Colombians consuming diets of lower quality and depleted in fiber have consistently been associated with dysbiosis and disease [36,37]. *Escherichia coli* is a facultative anaerobe that causes metabolic endotoxemia and inflammation via translocation of lipopolysaccharide [51]. It has been involved in gut dysbiosis [52,53] and several of its strains cause a variety of human diseases, including severe diarrheal disease, urinary tract infections, meningitis, septicemia and colorectal cancer [54,55]. Enterotoxigenic strains of *Bacteroides fragilis* have been associated with diarrhea [56] and colorectal cancer [55], and *Prevotella melaninogenica* has been found to be associated with periodontal abscesses, endocarditis and gynecological infections [57].

Reduced fiber intake and the growing availability of ultraprocessed foods are common themes in populations transitioning from traditional to Westernized lifestyles, as is the case of Colombians [58] and many other human populations [59,60,61]. We indeed observed that young individuals in our cohort (18–40 years) consumed diets of lower quality and increased intake of ultraprocessed foods compared with middle-aged adults (41–62 years), suggesting that a nutritional transition is occurring. It has been argued that changes in dietary patterns following industrialization and economic development have been too fast for our microbiome to adapt [62]. As the gut microbiome is intimately tied to human health [63], maladaptations in this community can severely contribute to the growing burden of noncommunicable diseases [62] that disproportionately affect developing countries [61,64]. Here, we showed that dietary patterns leave imprints in the gut microbial community. Our results are important for understanding the emergence of diet-driven dysbiosis in the context of broader lifestyle changes. We anticipate that the association between “Westernized” diets, low in fiber and plant polysaccharides and high in fats and sugars, and an inflammatory gut microbiota will be stronger as the nutritional transition progresses, with deleterious consequences for the generations to come.

Our study has several strengths. As we showed, the analyzed population was genetically different to well-studied Americans and Europeans [65] and harbored a different baseline gut microbiota [36,66], extending diet-microbiota interactions to poorly explored human populations. We inquired about the diet of community-dwelling adults of both sexes, analyzed a large sample size and measured several covariates that allowed us to adjust statistical analyses for potential confounders. Importantly, we employed several statistical analyses including a machine-learning algorithm that identified associations between diet and community structures, not individual microbes. This is particularly relevant as microbes cannot be taken as isolated entities, but form communities that interact, compete and cross-feed. The association of a particular microbe with a given nutritional variable may be low, but the signal at the community level (i.e., the microbiota) is what these methods detect. However, it is important to acknowledge its limitations. This is a cross-sectional study, preventing inference into causal relationships. Diet information was collected only once for the majority of participants and was assessed through 24-HDR interviews that tested the participants’ ability to remember what they ate [67]. These interviews may be subject to reporting bias and measurement errors. Also, the nutritional databases used as references overlooked information about foods and nutrients that might impact the gut microbial community, including phenolic compounds, different types of fermentable fibers and food additives, as underlined previously [68].

Recent studies have highlighted the personalized nature of diet-microbiota associations [68,69,70], and trends in personalized nutrition suggest that diet can be used to modulate the microbiome [71]. Johnson et al. [68] elegantly showed that similar foods have different effects on different subject’s gut microbiomes. Despite this insightful evidence, we and others have observed consistent and reproducible associations between diet and gut microbes that encompass populations with diverse geographic origins, diets and lifestyles. Our results add to the growing evidence that diet is a strong driver of the gut microbial community and highlight specific associations between diet and microbiota structures that suggest common pathways through which gut microbes respond to the human diet. More generally, we do not consider that there is opposition between personalized and generalized responses of the microbiota to the diet. These two responses are extremes of a continuum. Analyzed at fine levels (e.g., microbial strains, OTUs, amplicon sequence variants and enzymes), the response of the microbiome to a certain food or nutrient seems very specific and personalized [68,72,73]. However, on a broader level (e.g., higher taxonomic ranks and broad metabolic pathways), we have underlined the emergence of general trends. The astonishing functional redundancy of bacteria lies beneath these common responses, as changes in the phylogenetic composition of the microbial community can take place without significant shifts in its metabolic capabilities [68,74,75]. As more studies arise in community dwellers and diverse populations, more of the general principles guiding microbial responses to diet will be evidenced, thus serving as important hypothesis generators that could be tested in controlled dietary interventions.

## 5. Conclusions

This study expands our understanding of the relationship between dietary intake and gut microbiota. We provide (1) evidence that diet is strongly associated with the gut microbial community, (2) extend results to an understudied human population and (3) highlight associations that suggest common pathways through which the human gut microbiota connects to diet.

## Figures and Tables

**Table 1:** Diet intake in the studied population grouped by sex and age group. Average and standard deviation values (within parentheses) are shown.

	Males		Females		
	18–40 Years	41–62 Years	18–40 Years	41–62 Years	
	(*n* = 98)	(*n* = 114)	(*n* = 109)	(*n* = 120)	*p*-Value ^1^
**Diet quality**					
HEI ^2^	37.3 (7.44)	40.4 (8.80)	39.1 (9.64)	41.7 (9.18)	/***
GABA ^3^	22.3 (10.0)	27.6 (10.6)	24.1 (10.7)	28.6 (10.0)	NS/***
Ultraprocessed foods (%) ^4^	34.7 (16.2)	30.9 (15.8)	39.0 (18.6)	34.7 (15.6)	*/**
**Food groups**					
Dairy (g)	172 (196)	183 (196)	186 (240)	201 (168)	NS/NS
Meats (g)	170 (122)	136 (94.3)	113 (81.7)	86.5 (60.8)	***/***
Eggs (g)	40.8 (59.9)	39.0 (43.7)	38.2 (45.0)	32.9 (47.1)	NS/NS
Beans (g)	78.4 (185)	38.0 (94.4)	29.1 (63.1)	29.9 (69.3)	*/.
Nuts (g)	1.53 (7.85)	2.91 (12.4)	2.37 (9.45)	3.51 (26.8)	NS/NS
Fruits (g)	200 (238)	232 (243)	171 (197)	221 (257)	NS/.
Vegetables (g)	72.9 (80.8)	97.6 (133)	74.2 (75.3)	105 (106)	NS/**
Cereals (g)	350 (164)	333 (201)	230 (152)	203 (137)	***/NS
Tubers (g)	220 (208)	174 (180)	136 (147)	90.7 (113)	***/**
Fats (g)	33.6 (29.7)	25.6 (29.9)	22.3 (26.7)	14.2 (18.2)	***/**
Sugars (g)	339 (348)	213 (239)	178 (203)	141 (202)	***/***
**Nutrients**					
Calories (kcal)	2240 (389)	2030 (487)	1830 (347)	1670 (316)	***/***
Macronutrients					
Carbohydrates (g)	305 (53.4)	286 (76.5)	248 (44.9)	232 (50.3)	***/**
Proteins (g)	81.5 (12.4)	76.6 (11.5)	70.4 (9.65)	67.5 (10.5)	***/***
Fats (g)	72.0 (15.6)	62.9 (16.1)	62.0 (14.0)	55.0 (12.3)	***/***
SFA (g) ^5^	28.9 (7.35)	24.6 (7.32)	24.6 (7.11)	21.7 (5.52)	***/***
MUFA (g) ^6^	24.4 (4.70)	21.8 (4.95)	21.5 (4.69)	19.3 (4.14)	***/***
PUFA (g) ^7^	14.7 (4.74)	12.4 (4.68)	11.9 (4.00)	9.84 (3.44)	***/***
Cholesterol (mg)	354 (37.9)	346 (34.7)	336 (32.8)	329 (35.0)	***/*
Fiber (g)	19.4 (5.09)	18.6 (4.87)	16.3 (4.46)	16.7 (5.26)	***/NS
Micro-nutrients					
Ca (mg)	664 (269)	632 (235)	581 (230)	623 (213)	./NS
p (mg)	1150 (247)	1070 (235)	961 (213)	931 (182)	***/*
Total Fe (mg)	14.5 (2.22)	13.9 (2.19)	12.8 (1.82)	12.6 (1.87)	***/NS
Na (mg)	1420 (389)	1270 (384)	1280 (367)	1160 (292)	***/***
K (mg)	3410 (767)	3300 (870)	2820 (781)	2750 (738)	***/NS
Mg (mg)	269 (54.2)	257 (62.1)	221 (46.1)	214 (46.9)	***/.
Zn (mg)	10.7 (0.608)	10.6 (0.596)	10.2 (0.694)	10.1 (0.659)	***/*
Cu (mg)	2.16 (1.16)	1.87 (1.13)	1.41 (0.667)	1.36 (0.583)	***/.
Mn (mg)	3.36 (0.489)	3.26 (0.629)	2.99 (0.498)	2.93 (0.492)	***/NS
Vitamin A (RE)	836 (213)	834 (177)	756 (148)	798 (148)	***/NS
B1 (mg)	1.38 (0.673)	1.17 (0.318)	1.06 (0.296)	1.03 (0.274)	***/**
B2 (mg)	1.97 (0.842)	1.80 (0.444)	1.66 (0.440)	1.62 (0.453)	***/.
B3 (mg)	19.7 (7.43)	17.5 (5.16)	15.2 (3.71)	14.1 (3.22)	***/***
B5 (mg)	5.79 (2.13)	5.37 (1.41)	4.53 (0.867)	4.51 (1.05)	***/NS
B6 (mg)	1.56 (0.798)	1.56 (0.641)	1.51 (0.675)	1.40 (0.532)	./NS
B9 (folate) (µg)	378 (78.1)	359 (73.6)	332 (58.1)	328 (64.0)	***/.
B12 (mg)	7.28 (0.376)	7.21 (0.375)	7.11 (0.350)	7.02 (0.373)	***/*
Vitamin C (mg)	171 (68.5)	183 (62.0)	150 (58.3)	162 (59.9)	**/*

^1^*p*-values from ANOVA testing differences by sex (left) and age group (right). NS = *p* > 0.10; = *p* < 0.10; * = *p* < 0.05; ** = *p* < 0.01; *** = *p* < 0.001.; ^2^ Adapted version of the Healthy Eating Index (HEI), see Methods; ^3^ Index based on the Colombian Food-Based Dietary Guidelines (GABA), see Methods; ^4^ Percentage of consumed calories contributed by ultraprocessed foods; ^5^ SFA = saturated fatty acids; ^6^ MUFA = monounsaturated fatty acids; ^7^ PUFA = polyunsaturated fatty acids.

## Supplementary Materials

The following are available online at https://www.mdpi.com/2072-6643/12/10/2938/s1, Table S1: Number of recruited individuals in the original cohort by city, sex, age group, BMI and socioeconomic status, Table S2: Adapted HEI-2015, components and scoring standards, Table S3: GABA index, food group and values standard for a maximum score, Table S4: Adequacy of nutrients and food groups by sub-groups of age and sex, Table S5: Random forest models associating the most abundant OTUs and diet features.
